# Enhanced soft error resilience of NBTI-tolerant dual mode Schmitt trigger circuit

**DOI:** 10.1038/s41598-025-11595-6

**Published:** 2025-07-16

**Authors:** Aryan Kannaujiya, Ambika Prasad Shah

**Affiliations:** IC-ResQ Lab, Department of Electrical Engineering, Indian Institute of Technology Jammu, Jammu, J&K 181221 India

**Keywords:** Aging effects, CMOS, NBTI, Noise immunity, Schmitt trigger, Soft error, Electrical and electronic engineering, Electronic and spintronic devices

## Abstract

A dual-mode inverting Schmitt trigger (DM-IST) circuit is presented. The proposed DM-IST circuit is configured with a single PMOS in pull-up and five NMOS in pull-down networks to reduce the effect of negative bias temperature instability (NBTI) on the circuit. Owing to NBTI, the increase in threshold voltage ($\mathrm {V_{th}}$) shift for the proposed DM-IST circuit is only 1.43$\times$ in comparison to the conventional Schmitt trigger after the stress time of 10 years. The $\mathrm {V_{th}}$ and leakage current of DM-IST is estimated mathematically with respect to conventional design. It is observed that the proposed circuit has a hysteresis width of 1.39$\times$ improved than the conventional Schmitt trigger. Further, this work has been extended to analyze the effect of radiation by injecting a double exponential current. The critical charge, threshold current and area of the proposed DM-IST circuit is 2.89$\times$, 2.02$\times$ and 2.44$\times$ of the conventional circuit. Other performance metrics, such as dynamic power, leakage power, propagation delay, and power delay product are improved by 2.94$\times$, 1.89$\times$, and 1.60$\times$, and 4.71$\times$ respectively, compared to the conventional Schmitt trigger circuit. While Control unit is tuned from 0.6V to 1V by varying input and supply voltage together, the critical charge and hysteresis width is observed. All these analysis reveal that the proposed DM-IST circuit is more efficient, with enhanced noise immunity and the capability for reliable operation in radiation-induced environments over the long term. All simulation work has been performed using 40 nm UMC technology using the Cadence Virtuoso tool.

## Introduction

As the technology node is scaling down, the reliability and power consumption of the circuits are considered conflicting metrics. Bias temperature instability (BTI) is the severe reliability challenge for new generation Complementary Metal–Oxide–Semiconductor (CMOS) technology^1^. It causes performance degradation in P-channel MOS (PMOS) and N-channel MOS (NMOS) referred to as negative BTI (NBTI) and positive BTI (PBTI). In comparison to NBTI in PMOS and PBTI in NMOS, NBTI/NMOS and PBTI/PMOS have a high influence on the circuit lifetime. NBTI and PBTI are caused by single oxide defects that are charged under normal working circumstances. Higher temperatures increase the chances of trapping charges in both devices and may raise the transistor threshold voltage ($\mathrm {V_{th}}$) and reduce transconductance and mobility. Long-term degradation of device performance due to NBTI and PBTI may result in timing errors, lower noise immunity and increased short-circuit glitches in logic and memory^2^. In addition to being vulnerable to Bias Temperature Instability (BTI), aggressively scaled CMOS devices are becoming more prone to radiation-induced faults, which further deteriorate the circuit’s noise immunity^3^. Specifically, single-event effects (SEE) caused by radiation primarily result from the reduction in supply voltages and node capacitances. A Single Event Upset (SEU) is the most common type of Single Event Effect (SEE) in storage elements such as latches and memory cells, potentially causing a change in their logic state. In contrast, a Single Event Transient (SET) occurs in combinational circuits, generating a voltage glitch that can propagate through logic blocks, potentially leading to soft errors^4^. A glitch is a temporary, unintended voltage spike or drop, essentially a momentary logic-level transition that does not represent a real change in input but is caused by some disturbance. In the context of soft errors, a glitch is typically caused by a single-event transient (SET), where a high-energy particle strike induces a transient current at a sensitive node (e.g., a reverse-biased pn-junction), momentarily short circuit glitch in logic levels^5,6^. Focusing on its impacts, in digital circuits especially in logic gates, inverters, or memory elements, a glitch can propagate through combinational logic and be latched by a flip-flop or latch if it occurs close to a clock edge, causing a single-event upset (SEU). Moreover it disrupt signal integrity, resulting in incorrect data processing or output. In Schmitt Trigger (ST) circuits, SET-induced glitches can cause false triggering due to their hysteresis behavior because STs are sensitive to sharp transitions. Short circuit glitch degrade the noise immunity of ST. It temporarily affect the switching threshold or output, depending on node sensitivity. In the present work the short circuit glitch caused by SET is clearly detrimental because it causes logic flipping for short period of time as well as it introduces noise to the signal and impact the noise immunity. The occurrence of a glitch indicates vulnerability in the transient response of circuit. Alpha particles and neutrons from cosmic ray radiation can cause soft errors, impacting circuit performance in both terrestrial and space environments^3^. Specifically, in modern CMOS technology, research has shown that neutron-induced alpha particle generation plays a significant role in overall soft error rates.

Moreover, direct ionization from secondary protons can affect circuits operating at low voltages, which exhibit a reduced critical charge ($\mathrm {Q_{crit}}$)^6^. The decrease of the critical charge with supply voltage reduction has been studied in detail by Hao et. al^7^ for the latch circuit. The critical charge is the least amount of charge accumulated at the sensitive node during a particle strike that is sufficient to maintain the input and output states unchanged of the circuit. Furthermore, Negative Bias Temperature Instability (NBTI) exacerbates the soft error rate (SER) by reducing the critical charge ($\mathrm {Q_{crit}}$) over time under stress. Consequently, a substantial increase in SER is anticipated in deep submicron technologies. Furthermore, NBTI stress exacerbates the degradation of conventional CMOS inverters, leading to a scenario where a low-impedance path forms between the supply and ground during slow high-to-low (H-L) and low-to-high (L-H) transitions^8^. Employing a circuit configuration with a single PMOS and predominantly NMOS transistors can mitigate NBTI-related issues. However, this approach has certain limitations, including decreased output voltage levels, poor noise immunity, and the presence of a direct current path when both the Pull-Down Network (PDN) and Pull-Up Network (PUN) are simultaneously active. In deep sub-micron technologies, digital circuits exhibit a reduced static noise margin due to the threshold voltage ($\mathrm {V_{th}}$) of transistors not scaling proportionally with the supply voltage^9^. As a result, the input signal is more susceptible to external radiation, making noise a critical concern for IC designers^10^. A viable approach to mitigate the effects of slowly varying input signals and improve noise margin involves employing Schmitt trigger (ST) circuits^10–12^. Schmitt Trigger circuits enhance noise immunity and effectively process slowly varying input signals^13^. The switching threshold voltage of an ST circuit varies based on the direction of the input signal transition, which is determined by the hysteresis in its transfer characteristics. As a result, the ST output remains stable and does not immediately react to input fluctuations that are smaller than the threshold difference. This significantly improves the noise resilience of ST circuits compared to conventional CMOS designs. The conventional ST presented in Ref.^14^ fails to function properly at reduced transistor sizes. Dokic et al.^15^ proposed an ST design utilizing a single NMOS feedback, which exhibits high sensitivity to process variations. Anselmo et al.^16^ explored feedback configurations involving series and parallel transistor associations, where larger transistor implementations result in increased delay and significant area overhead. Fernandes et al.^17^ introduced a three-stage inverter-based design with feedback implemented without a transistor, leading to unstable hysteresis characteristics.

Furthermore, Moghaddam et al.^18^ and Toledo et al.^19^ have analyzed the performance of ST circuits under process, voltage, and temperature (PVT) variations, providing an in-depth discussion on the advantages and limitations of ST inverters. In addition to enhancing noise margins and exhibiting robustness against process variations, ST-based circuits have been extensively studied for their effectiveness in improving soft error resilience.For instance, the hysteresis characteristic of the ST circuit is leveraged to suppress soft error transient pulses in circuits^20,21^. Meanwhile, Jooq et al.^20^ introduce a hardened latch incorporating an ST-based design to enhance resilience against single-event effects, specifically addressing multiple-node upsets. Finally, An analysis of ST inverter chains under subthreshold conditions was conducted by Gadlage et al.^22^, demonstrating that improved soft error hardening and greater tolerance to single-event effects are achieved in ST-based circuits compared to conventional designs. While prior research in^23^ has shown that ST-based designs effectively enhance noise margins and mitigate soft errors, the influence of NBTI on ST circuits has not yet been explored. More recently, Ref.^24^ highlighted that ST circuits exhibit greater susceptibility to NBTI compared to CMOS inverters, primarily due to the presence of three PMOS transistors in the ST design, as opposed to a single PMOS transistor in a standard CMOS inverter. To address this limitation, they have introduced a more efficient circuit designed to mitigate the adverse effects of NBTI, referred to as the NMOS-only Schmitt Trigger with Voltage Booster (NST-VB). This design employs a rail-to-rail configuration consisting solely of NMOS transistors, significantly minimizing the influence of NBTI on circuit performance. The NST-VB architecture enhances noise margin through feedback connections and ensures a more robust output, attributed to the integration of the voltage booster circuit. In Ref.^25^, the fundamental principles of the NST-VB circuit were presented; however, its effectiveness in enhancing soft error resilience under the influence of BTI ageing has not been assessed.

All the previously reported Schmitt Triggers (STs) enhance the circuit’s noise margin but are susceptible to NBTI due to the high number of PMOS transistors in the pull-up network (PUN). To address these challenges, a Dual-Mode Inverting Schmitt Trigger (DM-IST) has been proposed, incorporating a single PMOS transistor along with feedback and diode connections into the conventional ST design. Furthermore, a comprehensive analysis has been conducted on delay, dynamic power, leakage power, and $\mathrm {V_{th}}$ shift variations over a 10-year period for all considered circuits. We initially assess the susceptibility to soft errors by comparing the proposed design with functionally equivalent counterparts, focusing on variations in critical charge under different stress durations and supply voltage levels. Specifically, we evaluate and compare the critical charge sensitivity across all considered circuits, followed by an analysis of threshold voltage degradation under varying stress conditions and supply voltages. Further, all these circuitry are configured with a single operation. In this work, we are proposing a Dual-Mode Inverting Schmitt Trigger (DM-IST) with additional transistors operated by the external voltage to decide the mode of operation. DM-IST has been examined for NBTI test for ten years. Additionally, in relation to this research work, the preliminary work is presented in Ref.^11^ and the presented DM-ST is extended for testing for radiation hardening effect along with NBTI effect. The key contributions of this work are as follows:

- To utilize the DM-IST circuit for dual purpose. First as an inverting Schmitt trigger circuit and as an inverter using a control circuit.
- Computation of mathematical modelling to estimate high to low ($\mathrm {V_{HL}}$) and low to high ($\mathrm {V_{LH}}$) threshold voltages.
- To assess DM-IST circuit reliability implications based on aging degradation, we have tested NBTI effect by applying stress/recovery mechanism for 10 years.
- Analysis of the effect of high radiation tolerance capability by evaluating critical charge at sensitive node.
- The noise immunity property has also been evaluated using the hysteresis window to assess its noise tolerance capability more effectively.

The proposed DM-IST circuit has a trade-off of process variation for leakage power and has a design complexity as it has extra control which add up area overhead, and if not properly designed, the control unit may introduce unintended feedback effects, affecting the robustness of the circuit. The work is organized into four distinct sections. “Introduction” section provides a comprehensive discussion of the ST, while “Proposed circuit configuration” section focuses on the proposed approach. In “Simulation results and discussions” section, we present the simulated results, and the concluding remarks are summarized in “Conclusion” section.

**Fig. 1:**
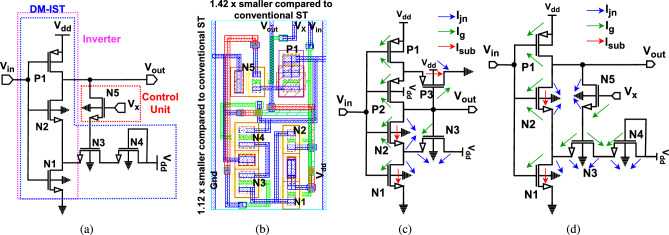
Proposed dual mode domino-based Schmitt trigger (DM-IST) circuit (**a**) Schematic with control module, (**b**) Optimized layout for area estimation, and leakage current estimation of (**c**) Conventional inverting Schmitt Trigger, (**d**) proposed DM-IST.

## Proposed circuit configuration

The proposed dual-mode inverting Schmitt trigger circuit (DM-IST) is inspired by a conventional Schmitt trigger circuit^14,26,27^. The proposed DM-IST is designed with six transistors, featuring one PMOS in the pull-up network and five NMOS in the pull-down network, as illustrated in Fig. 1a. DM-IST is designed with only one PMOS to reduce the major NBTI side effects. Only P1 is acting as a main pull up transistor and N1, N2, N3, N4 and N5 are the main pull down transistors. These transistors are kept as small as possible. The size of pull down network are N1 and N2 are of same size, N3 is 2.22$\times$ of N1 and N4 is same as N3. Further N5is 0.66$\times$ of N1 where as pull up network P1 is 1.33$\times$ of N1 respectively. These transistor sizing is estimated to maintain the equal rise and fall time, i.e., $\mathrm {\tau _{plh}}$ and $\mathrm {\tau _{phl}}$. The DM-IST functions both as an inverter and a noise-immune Schmitt trigger. The transistor N5 acts as a control unit, enabling the dual-mode operation of the DM-IST. When an external voltage $\mathrm {V_{x}}$ is set to 0 (low logic level), the DM-IST operates as an inverter; conversely, when $\mathrm {V_{x}}$ is set to 1 (high logic level) then positive feedback transistor get active which make the DM-IST act as an inverting Schmitt trigger. Additionally, the transistor N4 plays a crucial role in maintaining the current holding capability due to its diode-connected circuitry, which is part of the positive feedback loop involving N3. N4 also enhances the robustness of circuit against variations in the supply voltage. The dual functionality of the DM-IST is essential because traditional Schmitt triggers have a constrained input voltage range where they function optimally. If the input signal exceeds this range, the Schmitt trigger may fail to deliver the expected output. This limitation is particularly challenging when processing signals with a wide range of voltage levels. Furthermore, the dual-mode design of DM-IST addresses these challenges, offering greater versatility in handling diverse signal conditions.

**Fig. 2:**
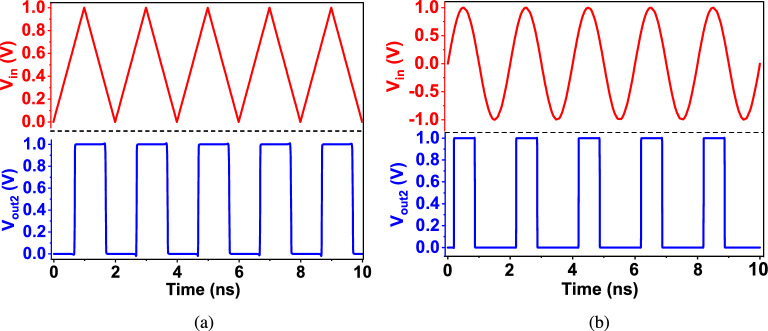
Conversion into a true square wave of (**a**) triangle wave and (**b**) sine wave when used as an inverter.

Since a traditional inverter is not appropriate for all digital signal processing tasks, leading to undesired outcomes due to a narrow operating voltage range. Thus, DM-IST can also be configured to act as an inverter for specific purposes by keeping $\mathrm {V_{x}=0}$, which handles the above challenges. Whenever high noise application is required, then it can be configured as ST by keeping $\mathrm {V_{x}=1}$. For better area understanding, the layout of DM-IST has been shown in Fig. 1b. The area of reported circuits^14–17^ are 2.44$\times$, 0.82$\times$, 2.25$\times$ and 1.10$\times$ respectively of proposed DM-IST circuit and tabulated in Table 3. To estimate the $\mathrm {V_{LH}}$ and $\mathrm {V_{HL}}$, i.e., low to high and high to low threshold voltages, numerical has been evaluated.

### Analytical model for $\mathrm {V_{LH}}$ and $\mathrm {V_{HL}}$

#### Calculation of $\mathrm {V_{LH}}$

For calculation of $\mathrm {V_{LH}}$ consider $\mathrm {V_{in}=0}$, and $\mathrm {V_{out}}=1$, We begin to find $\mathrm {V_{LH}}$. At this point, the output is at a high voltage, say $\mathrm {V_{dd}}$. The input is raised from 0 V toward $\mathrm {V_{dd}}$. At the nearby point of $\mathrm {V_{LH}}$, both transistors P1 and N2 are in their saturation regions of operation. The effect of other transistors is negligible during transition form 0$\rightarrow$1 so they are ignored. Hence, we have

1 $$\begin{aligned} \mathrm {I_{P1,sat} = I_{N2,sat}}. \end{aligned}$$

Let $\mathrm {\sqrt{K_{N2}/K_{P1}}}$ are transconductance ratio of N2 and P1, and $\mathrm {K = \mu _{0}\cdot C_{ox}\cdot {W}/{L}}$, hence

2 $$\begin{aligned} \mathrm {K_{P1}\left( V_{in}-V_{dd}-\left| V_{tp} \right| \right) ^{2}=K_{N2}\left( V_{in}-V_{y}-V_{tn} \right) ^{2}}. \end{aligned}$$

On evaluating Eq. (2), and replacing $\mathrm {V_{in}=V_{LH}}$, we obtained final expression of $\mathrm {V_{LH}}$

3 $$\begin{aligned} \mathrm {V_{LH} = \frac{\sqrt{\frac{K_{N2}}{K_{P1}}}\left( V_{y}-V_{tn} \right) +V_{dd}+V_{tp}}{1-\sqrt{\frac{K_{N2}}{K_{P1}}}}}, \end{aligned}$$

where $\mathrm {V_{y}}$ is the voltage across the source and drain of transistors N2 and N1. Since the current in transistors N1 and N2 are the same thus,

4 $$\begin{aligned} & \mathrm {I_{N2,sat} = I_{N1,sat}}, \end{aligned}$$

5 $$\begin{aligned} & \mathrm {K_{N2}\left( V_{in}-V_{tn}-V_{y}\right) ^{2}=K_{N1}\left( V_{in}-V_{tn}\right) ^{2}}, \end{aligned}$$

6 $$\begin{aligned} & \mathrm {V_{y} = V_{in}\left[ \sqrt{\frac{K_{N1}}{K_{N2}}}-1 \right] - V_{tn}\left[ \sqrt{\frac{K_{N1}}{K_{N2}}}+1 \right] }. \end{aligned}$$

#### Calculation of $\mathrm {V_{HL}}$

For calculation of $\mathrm {V_{HL}}$ consider $\mathrm {V_{in}=1}$, and $\mathrm {V_{out}}=0$, we begin to find $\mathrm {V_{HL}}$. At this point, the output is at a low voltage. The input is falling from 1 V toward the ground. At the nearby point of $\mathrm {V_{HL}}$, both transistors P1 and N3 are in their saturation regions of operation. The effect of other transistors is negligible during transition form 1 $\rightarrow$ 0, so they are ignored. Hence, we have

7 $$\begin{aligned} & \mathrm {I_{P1,sat} = I_{N3,sat}}, \end{aligned}$$

8 $$\begin{aligned} & \mathrm {\mathrm {K_{P1}\left( V_{dd}-V_{in}-\left| V_{tp} \right| \right) ^{2} = K_{N3} \left( V_{in}-V_{y}-V_{tn} \right) ^{2}}}. \end{aligned}$$

**Fig. 3:**
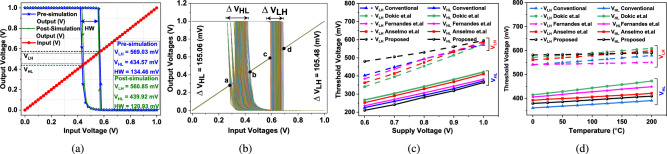
(**a**) Transfer characteristic forming hysteresis loop when used as DM-IST is used as ST, (**b**) MC simulation with 5000 iterations of the hysteresis loop, and rate of change of threshold voltage with varying (**c**) supply voltages and (**d**) temperature.

On evaluating Eq. (8) and replacing $\mathrm {V_{in}=V_{HL}}$, the final expression of $\mathrm {V_{HL}}$ is given below.

9 $$\begin{aligned} \mathrm {V_{HL} = \frac{\sqrt{\frac{K_{N3}}{K_{P1}}}\left( V_{tn}+V_{y} \right) +V_{dd} - \left| V_{tp} \right| }{1+\sqrt{\frac{K_{N3}}{K_{P1}}}}}. \end{aligned}$$

Therefore, the two switching threshold voltages of the proposed DM-IST circuit are given by Eqs. (3) and (9). The optimal hysteresis width can potentially be achieved by properly sizing the transistors in the circuit. The key determining factors of the HW are the physical characteristics and threshold voltages of transistors P1, N1, and N2. Expressions for $\mathrm {V_{LH}}$ and $\mathrm {V_{HL}}$ are derived for long-channel devices. The objective of this analytical model is to understand the hysteresis behavior of the proposed DM-IST circuit, with a primary focus on its threshold voltage switching characteristics.

## Simulation results and discussions

In this section, all evaluated results have been presented and discussed. For better understanding, DC and transient characteristics are studied in relation to the performance of all reported and proposed circuits in different operating conditions. Transient characteristics describe how a component or circuit reacts to rapidly changing conditions. Figure 2a shows the transition of the triangle wave into a square wave. Similarly, Fig. 2b shows the conversion of the sinusoidal wave into the square wave but has a difference in pulse width. The operating frequency of DM-IST is 500 MHz. The operating paramaters are varied from 0.6 to 1 V and $\mathrm {25\,^{\circ }C}$ to $\mathrm {125\,^{\circ }C}$ temperature. These operating volate and temmperature are based on work reported by Aryan et.al^10^.

### Noise immunity with hysteresis

DC characteristics shown in Fig. 3a exhibit a hysteresis loop, where the difference between $\mathrm {V_{LH}}$ and $\mathrm {V_{HL}}$ is hysteresis width (HW). The range of hysteresis width of reported circuits^14–17^ 1.39$\times$, 1.12$\times$, 1.36$\times$, and 1.28$\times$ respectively, that of DM-IST and the optimum HW of proposed DM-IST circuit is 134.46 mV at 1V and $\mathrm {25\,^{\circ }C}$. A larger HW window of DM-IST makes it more noise-immune than considered circuits. A wider hysteresis window reduces sensitivity to small fluctuations or noise in the input signal, preventing false triggering. Moreover, a wider hysteresis window ensures that the input must cross well-defined, voltage thresholds to change the output state. This spacing (voltage difference between the two switching thresholds) makes the circuit less responsive to minor voltage variations caused by noise or transient disturbances near the switching point. As a result, small fluctuations in the input signal are ignored, reducing the likelihood of unintended or rapid toggling of the output. Therefore, a wider hysteresis window enhances noise immunity and preventing false triggering, ensuring more reliable and stable operation of the circuit. It is because, if the hysteresis window is narrow, radiation induced glitches or small disturbances can cross the threshold and falsely trigger the output. Thus the voltage spike which comes under the wider hysteresis window is canceled out^28^. Thus, it ensures that the circuit remains in a stable state, reducing unintended switching due to minor variations in voltage. Moreover, DM-IST has transition due to addition of N-type of positive feedback but due to absence of P-type positive feedback it has no sharp transition. This absence is made to make it more NBTI resilient.

Since, Montecarlo (MC) simulations reveal the impact of process variations and uncertainties, thus 5000 MC of dynamic power has been performed for crystal comparison among considered circuits and proposed DM-IST. The industry-standard UMC 40 nm technology node has been used to simulate the proposed DM-IST including all reported considered circuits as shown in Fig. 3b where a, b, and c, d are the ranges of $\mathrm {V_{HL}}$ and $\mathrm {V_{LH}}$. Table 1 depicts the MC range for all circuits where DM-IST has less range of variation of thresholds, thus DM-IST is robust to process variation. Further, on running MC simulation, it is observed that the $\mathrm {V_{HL}}$ has high variation than $\mathrm {V_{LH}}$. It is none other than N-type positive feedback with high randomness. For better understanding, we have varied the $\mathrm {V_{HL}}$ and $\mathrm {V_{LH}}$ with supply voltage as shown in Fig. 3c. On increasing supply voltage from 0.6 to 1 V, the $\mathrm {V_{HL}}$ increases linearly for all reported as well as proposed DM-IST circuits under the influence of supply voltage. Whereas, $\mathrm {V_{HL}}$ has high impact on reported circuits^14–17^ but has less impact on $\mathrm {V_{HL}}$ on proposed DM-IST circuit. It is because the proposed DM-IST has only one PMOS transistor in the pull-up the network. Moreover, Fig. 3d shows the variation of $\mathrm {V_{HL}}$ and $\mathrm {V_{LH}}$ as a function of temperature from $\mathrm {25\,^{\circ }C}$ to $\mathrm {125\,^{\circ }C}$. There is a gradual increase in the DM-IST circuit than that of the reported circuit^14–17^. It is because at higher temperatures, mobility reduction is due to increased phonon scattering, which becomes more dominant. This reduction in mobility affects the current more than it does the threshold voltage, which makes $\mathrm {V_{HL}}$ and $\mathrm {V_{LH}}$ relatively stable with respect to temperature for DM-IST.

**Fig. 4:**
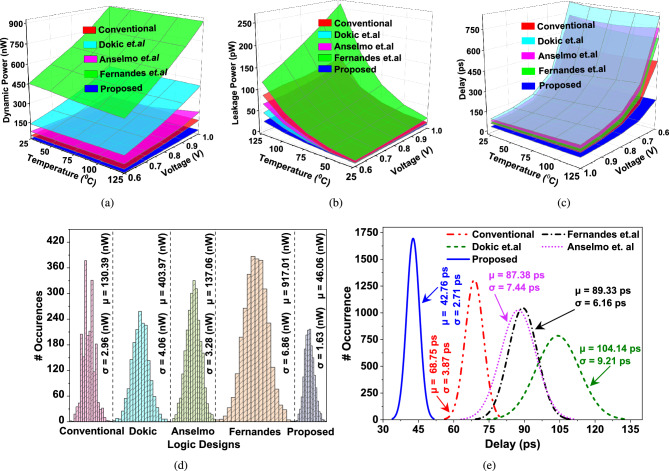
Effect of supply voltage and temperature variations on (**a**) dynamic power, (**b**) leakage power, (**c**) propagation delay, and Monte Carlo simulation with 5000 iterations of (**d**) dynamic power, (**e**) propagation delay of different Schmitt trigger circuits.

**Table 1:** Process variations of HW of proposed DM-IST and reported circuits @1 V and $\mathrm {25\,^{\circ }C}$.

Logic designs	$\varvec{\mathrm {\Delta V_{HL}}}$ (mV) (a–b)	$\varvec{\mathrm {\Delta V_{LH}}}$ (mV) (c–d)
Conventional^14^	201.98–510.09	528.26–717.63
Dokic et al.^15^	266.63–446.01	506.71–689.44
Anselmo et al.^16^	240.51–474.22	541.03–709.48
Fernandes et al.^17^	180.56–526.88	490.34 - 721.81
**Proposed DM-IST**	282.21–437.08	588.75–693.41

**Table 2:** Comparison of performance parameters of proposed DM-IST: pre and post layout simulation @1 V and $\mathrm {25\,^{\circ }C}$.

Performance parameter	Pre - layout simulation	Post - layout simulation
HW (mV)	134.46	120.93
Leakage power (pW)	5.03	9.35
Dynamic power (nW)	43.98	57.09
Propagation delay (ns)	42.55	54.23
PDP (aJ)	18.71	30.95

### Power and delay analysis

For the fair comparison, we have considered 12 fF of load capacitance to analyze dynamic power ($\mathrm {P_{D}}$) and leakage power ($\mathrm {P_{L}}$) dissipation for all the considered circuits. On varying temperature from $\mathrm {25\,^{\circ }C}$ to $\mathrm {125\,^{\circ }C}$ and voltage from 0.6 to 1 V $\mathrm {P_{D}}$ and $\mathrm {P_{L}}$ increase with an increase in voltage and temperature, as shown in Fig. 4a. The dynamic power dissipation at 1 V and $\mathrm {25\,^{\circ }C}$ of considered circuits^14–17^ are 2.94$\times$, 9.12$\times$, 3.08$\times$ and 20.90$\times$ of proposed DM-IST circuit. Table 3 shows the optimum dynamic power considered and the DM-IST circuit. However, $\mathrm {P_{L}}$ dissipation in standby mode is shown in Fig. 4b. The optimum static power dissipation at 1 V and $\mathrm {25\,^{\circ }C}$ of considered circuits^14–17^ are 1.89$\times$, 1.54$\times$, 1.76$\times$ and 4.02$\times$ of proposed DM-IST. Table 3 tabulates the optimum static power of considered and DM-IST circuits. The propagation delay ($\mathrm {\tau _{D}}$) for the considered and proposed DM-IST has also been calculated. There is a sufficient reduction in delay on increasing voltage from 0.6 to 1 V. On the other hand on increasing temperature from $\mathrm {25\,^{\circ }C}$ to $\mathrm {125\,^{\circ }C}$, there is a gradual decrease in delay as shown in Fig. 4c. During the transition from low to high the capacitance associated with feedback of DM-IST starts charging and vice-versa. It causes a gradual delay in DM-IST. The delay of considered circuits^14–17^ are 1.60$\times$, 1.19$\times$, 2.03$\times$ and 1.70$\times$ respectively, higher as compared to DM-IST.

To understand process variation and process mismatch, we have run the Montecarlo simulation with 5000 iterations of dynamic power and propagation delay at 1 V and $\mathrm {25\,^{\circ }C}$. The process variation ($\mathrm {\sigma }$) and mean ($\mathrm {\mu }$) values of $\mathrm {P_{D}}$ for the conventional ST circuit^14^ are 1.81$\times$ and 2.83$\times$, respectively. For Dokic et al., these values are 2.49$\times$ and 8.77$\times$, while for Anselmo et al., they are 2.01$\times$ and 2.97$\times$. Fernandes et al. exhibit the highest values, with $\mathrm {\sigma }$ of 4.2$\times$ and $\mathrm {\sigma }$ of 19.90$\times$, all in comparison to the proposed DM-IST circuit. The optimum value of $\mathrm {P_{D}}$ of proposed DM-IST is $\mathrm {\sigma = 1.63\,nW}$ and $\mathrm {\mu = 46.06\,nW}$ at 1V and $\mathrm {25\,^{\circ }C}$. Figure 4d illustrates the optimum values of $\mathrm {P_{D}}$ for all circuits.

Further, the process variation ($\mathrm {\sigma }$) and mean $\mathrm {\mu }$ value of $\mathrm {\tau _{D}}$ for the conventional circuit^14^ are observed to be 1.43$\times$ and 1.59$\times$, respectively. For the design proposed by Dokic et al., $\mathrm {\sigma }$ and $\mathrm {\mu }$ exhibit 3.39$\times$ and 2.43$\times$, whereas Anselmo et al. report values of 2.75$\times$ and 2.04$\times$. Fernandes et al. demonstrate a $\mathrm {\sigma }$ of 2.27$\times$ and a $\mathrm {\mu }$ of 2.08$\times$, all evaluated relative to the proposed DM-IST circuit. The optimum value of $\mathrm {\tau _{D}}$ of DM-IST is $\mathrm {\sigma = 2.71\,ps}$ and $\mathrm {\mu = 42.76\,ps}$ at 1V and $\mathrm {25\,^{\circ }C}$. Figure 4e illustrates the optimum values of $\mathrm {\tau _{D}}$ for all considered and proposed circuits. There is less process variation observed in $\mathrm {P_{D}}$ and $\mathrm {\tau _{D}}$ of DM-IST, thus the proposed DM-IST is process variation resistant and reliable operation.

**Fig. 5:**
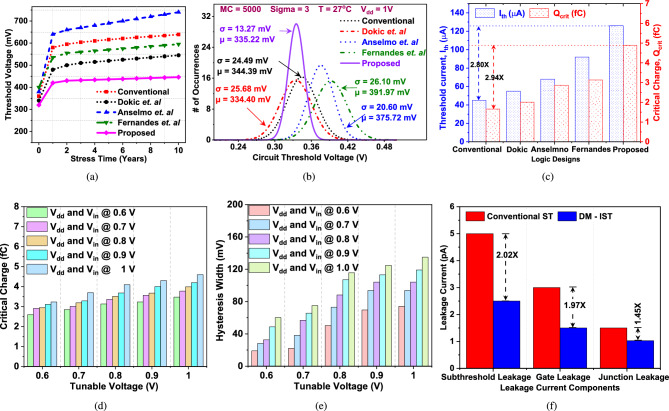
(**a**) Rate of change of threshold voltage with varying supply voltages, (**b**) 5000 Monte Carlo iterations on circuit threshold voltages, (**c**) relation between critical charge and peak current of different Schmitt trigger circuits, and effect of tunable voltage on (**d**) critical charge, (**e**) Hysteresis width and (**f**) Leakage current components of conventional ST in relation to proposed DM-IST.

### Analysis of NBTI effect

The PMOS transistor (P1) is susceptible to NBTI when it is under stress at elevated temperatures. When $\mathrm {V_{th}}$ is low, P1 is turned on, and this condition causes negative gate stress for an extended period of 10-year stress. This increase in $\mathrm {V_{th}}$ leads to slower switching behaviour, decreasing drain current and transconductance, and a higher input voltage required to turn P1 ON, affecting the overall operation of the proposed DM-IST. Due to NBTI, the increasing $\mathrm {V_{th}}$ of P1 shifts the trigger points, meaning that the voltage levels required for switching from low to high and high to low will shift, potentially causing it to be less immune to noise and leading to reduced reliability in switching. The control unit, which includes transistors N5, is not directly suffering from NBTI in the same way transistors N1, N2, N3 and N4 are not affected by NBTI. It is because NBTI primarily affects PMOS transistors; due to this, NBTI stress causes unsaturated silicon dangling bonds, i.e., silicon-hydrogen (Si-H) bonds at the $\mathrm {Si-SiO_{2}}$ interface to break, creating positively charged interface traps that increase the $\mathrm {V_{th}}$ over time at elevated temperature^29^. This can result in less effective noise immunity and slower switching, impacting the reliability of the circuit. Furthermore, justification for PMOS arrangement in proposed DM-IST design is that, the PMOS transistor (P1) is active only during specific input conditions and is not continuously under negative gate bias. This results in a reduced duty cycle, allowing P1 to spend a significant portion of time in the recovery phase, thereby mitigating NBTI degradation^30^. Since PMOS devices are more susceptible to NBTI due to hole trapping and detrapping at the Si/$\hbox {SiO}_2$ interface, especially under negative bias and elevated temperature, minimizing the number of PMOS transistors is beneficial. During stress, energetic holes tunnel into the gate oxide and become trapped, increasing the threshold voltage over time and degrading performance. The situation is worsened by hydrogen-induced breaking of Si-H bonds, which creates interface traps and further shifts threshold voltage^31^. Additionally, due to the lower mobility of holes, PMOS devices exhibit longer dwell times at the interface, increasing the probability of trap generation. Therefore, the use of a single PMOS in the DM-IST structure not only reduces the overall stress but also simplifies NBTI management, enhancing the circuit’s long-term reliability^29^. We have used Cadence Virtuoso RelXpert Reliability Simulator for aging analysis for all simulations considering supply voltage $\mathrm {V_{dd}}$ of 1 V and the operating temperature (T) of $\mathrm {25\,^{\circ }C}$ to observe the accelerated aging effects.

For clear understanding of NBTI effect, We have calculated the $\mathrm {V_{th}}$ shift, keeping temperature $\mathrm {25\,^{\circ }C}$ and 1 V providing stress over 10 years using Reaction–Diffusion (RD) model for time estimation. The range of $\mathrm {V_{th}}$ shift of conventional^14^, Dokic et al.^15^, Anselmo et al.^16^ Fernandes et al.^17^ and proposed DM-IST are 360 mV to 639 mV, 340 mV to 545 mV, 380 mV to 740 mV, 400 mV to 595 mV and 320 mV to 446 mV respectively. There is less shift in $\mathrm {V_{th}}$ of proposed DM-IST than other considered circuits as shown in Fig. 5a. For the effective process variation and mismatch estimation, Montecarlo (MC) simulations of 5000 iterations reaveling the impact of process variations and uncertainties of $\mathrm {V_{th}}$ for reported and proposed DM-IST circuits has been illustrated in Fig. 5b. The process variation ($\mathrm {\sigma }$) and mean $\mathrm {\mu }$ value of conventional^14^, Dokic et al.^15^, Anselmo et al.^16^ Fernandes et al.^17^ are 1.84$\times$ and 1.02$\times$; 1.93$\times$ and 0.99$\times$; 1.55$\times$ and 1.12$\times$; 1.96$\times$ and 1.16$\times$ of proposed DM-IST circuit respectively. The optimum value of $\mathrm {\sigma }$ and $\mathrm {\mu }$ of proposed DM-IST are 13.27 mV and 335.22 mV at 1 V and $\mathrm {25\,^{\circ }C}$. Since there is less variability in $\mathrm {V_{th}}$ of DM-IST circuits because it has all NMOS transistors in the pull-down network except one PMOS in the pull-up network, making it robust against NBTI effect^29^.

**Table 3:** Performance comparison of different circuits @ 1 V and $\mathrm {25\,^{\circ }C}$.

Logic designs	Performance parameter							
	$P_\textrm{D}$(pW)	$P_\textrm{L}$(pW)	$\tau _\textrm{D}$ (ps)	PDP (aJ)	HW (mV)	$Q_{\textrm{crit}}$ (fC)	Area ($\upmu m^2$)	FoM
Conventional^14^	129.4	9.530	68.25	88.31	96.23	1.66	96	0.0398
Dokic et al.^15^	135.80	8.860	86.40	11.73	121.56	2.010	32.2	0.9825
Anselmo et al.^16^	401.30	7.760	103.2	41.41	98.87	2.860	88.40	0.1189
Fernandes et al.^17^	919.30	20.23	72.49	66.64	105.08	3.130	423.20	0.0519
Proposed DM-IST	43.98	5.030	42.55	18.71	134.46	4.890	39.20	1

### Leakage current estimation

In deep-submicron technology, leakage current is a significant issue that notably contributes to total power dissipation, particularly in circuits that remain idle for extended periods. In CMOS circuits, the primary components of short channel effects include subthreshold leakage current ($\mathrm {I_{sub}}$), junction leakage current ($\mathrm {I_{jn}}$), and gate leakage current ($\mathrm {I_{g}}$). Figure 1c,d illustrates the primary leakage current components in a conventional Schmitt trigger and proposed DM-IST circuits for low input logic. The proposed DM-IST circuit modifies the subthreshold leakage calculation by including additional terms, suggesting design optimizations that impact leakage mechanisms. The leakage current in the idle state of a conventional Schmitt trigger is evaluated below:

10 $$\begin{aligned} & \mathrm {I_{sub_{ST0}} = I_{sub_{M1}} + I_{sub_{M2}} + I_{sub_{M3}}}, \end{aligned}$$

11 $$\begin{aligned} & \mathrm {I_{g_{ST0}} = I_{gd_{M1}} + I_{gd_{M2}} + I_{gs_{M2}} + I_{gs_{M3}} + I_{gd_{M4}} \mathrm { + I_{gs_{M4}} + I_{gd_{M5}} + I_{gs_{M5}} + I_{gs_{M6}}}}, \end{aligned}$$

12 $$\begin{aligned} & \mathrm {I_{jn_{ST0}} = I_{jnd_{M1}} + I_{jnd_{M2}} + I_{jns_{M2}} + I_{jnd_{M3}} + I_{jns_{M3}}\mathrm {+ I_{jnd_{M6}}} }, \end{aligned}$$

13 $$\begin{aligned} & \mathrm {I_{Leakage_{ST0}} = I_{sub_{ST0}} + I_{g_{ST0}} + I_{jn_{ST0}} }. \end{aligned}$$

The leakage current in idle state for proposed DM-IST circuit has been evaluated below:

14 $$\begin{aligned} & \mathrm {I_{sub_{DM-IST0}} = I_{sub_{M2}} + I_{sub_{M3}}}, \end{aligned}$$

15 $$\begin{aligned} & \mathrm {I_{g_{DM-IST0}} = I_{gd_{M1}} + I_{gs_{M1}} + I_{gd_{M2}} + I_{gs_{M2}} + I_{gd_{M3}} + I_{gd_{M4}} + I_{gs_{M5}} + I_{gd_{M5}} + I_{gs_{M6}}}, \end{aligned}$$

16 $$\begin{aligned} & \mathrm {I_{jn_{DM-IST0}} = I_{jnd_{M1}} + I_{jnd_{M2}} + I_{jns_{M2}} + I_{jnd_{M3}} + I_{jnd_{M4}} + I_{jns_{M4}} + I_{jnd_{M5}} + I_{jns_{M5}} + I_{jnd_{M6}} + I_{jns_{M6}}}, \end{aligned}$$

17 $$\begin{aligned} & \mathrm {I_{leakage_{DM-IST0}} = I_{sub_{DM-IST0}} + I_{g_{DM-IST0}} + I_{jn_{DM-IST0}}}. \end{aligned}$$

The $\mathrm {I_{sub_{ST0}}}$, $\mathrm {I_{g_{ST0}}}$, and $\mathrm {I_{jn_{ST0}}}$, are 2.02$\times$, 1.97$\times$ and 1.45$\times$ that of DM-IST circuit. The The total leakage current of the proposed DM-IST Schmitt trigger is lower than the conventional ST design as shown in Fig. 5f, it indicates better leakage control. The optimum actual leakage current at 1 V and $\mathrm {25\,^{\circ }C}$ of conventional^14^, Dokic et al.^15^, Anselmo et al.^16^ Fernandes et al.^17^ and proposed DM-IST are 9.53 pA, 8.86 pA, 7.76 pA, 20.23 pA and 5.03 pA respectively.

### Analysis of radiation hardening

**Fig. 6:**
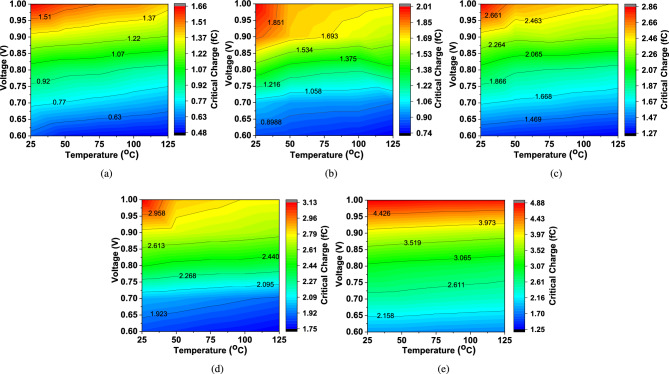
Effect of supply voltage and temperature variations on critical charge (**a**) Conventional^14^, (**b**) Dockic^15^, (**c**) Anselmno^16^, (**d**) Fernandes^17^, (**e**) Proposed DM-IST.

The incorporation of hysteresis in the circuit enhances noise margins, requiring a higher input voltage noise level to affect the output. Consequently, this results in an increased critical charge at the gate input, leading to improved soft error resilience. To evaluate the effectiveness of the proposed DM-IST circuit as a robust inverting Schmitt trigger against soft errors, particularly in comparison to other reported Schmitt Trigger configurations, it is important to note that single-event transients (SETs) are induced at sensitive nodes due to reverse-biased pn-junctions mechanism. This condition is most commonly found in the drain junctions of transistors that are in the OFF state. In such cases, the electric field across the junction reaches its peak value, as it is governed by the voltage drop across the junction, which is equal to $V_{dd}$. In a conventional CMOS inverter, two scenarios can arise. First, when the input is at logic 0 and the output is at logic 1, the NMOS transistor remains OFF, making its drain junction the vulnerable region. If a high-energy particle strikes this area, it can generate sufficient charge to induce a transient negative glitch, briefly switching the output from 1 to 0. Second, when the input is at logic 1 and the output is at logic 0, the PMOS transistor is OFF, and its drain junction becomes the sensitive node. An energetic particle impacting this region can lead to a transient positive glitch, momentarily causing the output to switch from 0 to 1^32^. The current transient pulse generated by an energetic particle is modeled by a double exponential current waveform, which is given as:

18 $$\begin{aligned} \mathrm {I_{inj}(t)=\frac{Q_{inj}}{\left( \tau _{f}-\tau _{r} \right) }\times \left( e^{\frac{t}{\tau _{f}}}-e^{\frac{t}{\tau _{r}}} \right) }, \end{aligned}$$

where $\mathrm {I_{peak}=\frac{Q_{inj}}{\left( \tau _{f}-\tau _{r} \right) }}$ and $\mathrm {I_{peak}}$is the peak value of the induced current. $\mathrm {Q_{crit}}$ is the minimum charge that is deposited by a particle strike to cause a short-duration glitch. A double exponential current signal has been used to represent the current pulse produced by ionizing particles at the sensitive node and is used to calculate the Critical charge ($\mathrm {Q_{crit}}$)^28^. Parameters $\mathrm {\tau _{f}}$ and $\mathrm {\tau _{r}}$ are fall time and rise time constant and fixed for the maximum current $\mathrm {I_{max}}$ that the current peak reaches during the transient. As explained in^9,33,34^, to have a fall time 50$\times$ higher than the rise time i.e, $\mathrm {\tau _{f} = 50\tau _{r}}$ has been set. According to Ref.^33^, we chose $\mathrm {\tau _{r}}$ of 10 ps, $\mathrm {\tau _{f}}$ of 500ps and $\mathrm {I_{max}}$ of 250 $\upmu$A. $\mathrm {Q_{crit}}$ can be expressed as:

19 $$\begin{aligned} \mathrm {Q_{crit}=\int _{0}^{T_{crit}}I_{inj}\left( t \right) \cdot dt}, \end{aligned}$$

where $\mathrm {I_{inj}\left( t \right) }$ is the injected current pulse at sensitive node. A clear understanding of critical charge and threshold current when a short circuit glitch occurs is shown in Fig. 5c. It is found that the is higher for the proposed DM-IST circuit than the other reported circuit^14–17^. In the proposed Schmitt trigger circuit, the critical charge ($\mathrm {Q_{crit}}$) is influenced by both temperature and supply voltage variations due to the intrinsic device characteristics and circuit topology of DM-IST. As the temperature increases from $\mathrm {25\,^{\circ }C}$ to $\mathrm {125\,^{\circ }C}$, the carrier mobility decreases, resulting in a reduction in transistor drive strength. This causes a slight decrease in the critical charge because the reduced drive strength leads to lower current through the transistors, thereby decreasing the amount of charge needed to flip the state of the circuit. On the other hand, when the supply voltage increases from 0.6 to 1 V, the overdrive voltage of the transistors also increases, leading to a substantial increase in current drive and switching speed. This significantly elevates the critical charge, as the higher current necessitates a larger amount of charge to disrupt the state of the circuit. Thus, while temperature variations lead to a marginal reduction in $\mathrm {Q_{crit}}$, voltage variation results in increased $\mathrm {Q_{crit}}$. The variation of $\mathrm {Q_{crit}}$ with respect to voltage and temperature of proposed and reported circuits have been shown in Fig. 6a–e. Increase in voltage cause significant effect on $\mathrm {Q_{crit}}$ rather than an increase in temperature. The observed high variations in the $\mathrm {Q_{crit}}$ charge due to varying voltage and temperature in reported^14–17^ circuits is because, as the voltage increases, the amount of charge stored in the node increases. Consequently, to cause a glitch in the circuit, more charge is needed to alter the state. Therefore, at higher voltages, the circuit becomes more resistant to radiation-induced errors because a higher amount of external charge (due to an ionizing particle) is required to cause a glitch, resulting in a higher critical charge^35^.

#### Effect of tunable voltage

The voltage $\mathrm {V_{x}}$ in the circuit serves as a control voltage that influences the operation of the control unit, particularly affecting the behaviour of N5 transistor. As $\mathrm {V_{x}}$ increases, it enhances the biasing of N5, making it more conductive and there by impacting the switching dynamics of the circuit. This adjustment allows the control unit to stabilize the output node $\mathrm {V_{out}}$ more effectively. As a result, the circuit becomes less susceptible to transient disturbances like those caused by radiation-induced charges. The control unit, through $\mathrm {V_{x}}$, manages the state of node more firmly, improving its ability to recover from external charge disruptions. Further, the effect of tunable voltage has been investigated for the proposed DM-IST. On tuning intermediate voltage $\mathrm {V_{x}}$ form 0.6 to 1 V, the critical charge increases as shown in Fig. 5d. Beyond 0.6 V, the DM-IST fails to work, so the range has been decided from 0.6 to 1 V. At 1 V of $\mathrm {V_{x}}$, $\mathrm {V_{dd}}$ and $\mathrm {V_{in}}$ the $\mathrm {Q_{crit}}$ is 4.89 fC. This happens because higher biasing of the control transistor N5 strengthens the resistance of the circuit to external disruptions, requiring a larger amount of charge to flip the node state of the output. The higher $\mathrm {V_{x}}$ effectively enhances the feedback strength and switching speed, making the circuit more robust and able to withstand higher radiation-induced charge without experiencing errors, leading to a significant increase in the critical charge.

Furthermore, As $\mathrm {V_{x}}$ increases, the stronger control over the switching dynamics effectively increases the separation between the switching thresholds during the upward and downward transitions of input voltage. This means that the circuit will require a higher voltage to switch from a low to a high state and a lower voltage to switch back, enlarging the hysteresis window. For slow-rising or slowly-varying input signals, a wider hysteresis prevents multiple switching events and ensures proper functioning. In radiation-hardened circuits, a larger hysteresis width helps mitigate transient effects caused by Single Event Transients (SETs) in harsh environments. $\mathrm {V_{x}}$ acts as a tunable parameter to adjust the hysteresis width and optimize the performance of circuit under varying operating conditions. On increasing $\mathrm {V_{x}}$ voltage from 0.6 to 1 V, the HW also increases as a function of $\mathrm {V_{dd}}$ and $\mathrm {V_{in}}$, shown in Fig. 5e. A wider hysteresis is beneficial in noisy environments, as it prevents small noise signals from causing unwanted toggling of the output, improving the stability of the circuit and noise immunity^36^. So, the Proposed DM-IST is highly suitable for noise environments and against accelerated ageing effects. Further, It is remarked that the variation in the critical charge with temperature for the proposed DM-IST is lower than for the compared circuits. Therefore, DM-IST is more soft error tolerant at higher temperatures. Moreover, it has less $\mathrm {V_{th}}$ shift over a period of time, it has less ageing effects on its performance parameters depending on specific applications and design goals.

### Post layout simulation

The proposed DM-IST has been tested for layout. The compact size of the DT-IST has been. There is minor degradation observed after. The performance parameter of pre and post layout simulation has been shown in Table 2. The hysteresis width, leakage power, dynamic power, propagation delay, are 0.89$\times$, 1.85$\times$, 1.29$\times$ and 1.22$\times$ of proposed DM-IST circuit. For area estimation and post layout simulation has been performed, the corresponding layout has been shown in the Fig. 1b. The optimum HW curve of pre and post has been updated in Fig. 3a. For energy calculation PDP has been shown in the Table 2 and it is 1.65$\times$ of proposed DM-IST circuit.

### Figure of merit

The figure of merit (FoM) is a metric used to evaluate the combined effect on performance parameters of considered circuits and depends on specific applications and design goals. The proposed FoM is reported in Eq. (20).

20 $$\begin{aligned} \mathrm {FoM=\frac{Q_{crit,N}\times ~HW_N}{P_{L,N}\times P_{PDP,N} \times V_{th,N} \times A_{N}}}, \end{aligned}$$

where $\mathrm {Q_{crit,N}}$ is critical chagre, $\mathrm {HW_N}$ is hysteresis width, $\mathrm {NM_{N}}$ is noise margin, $\mathrm {A_{N}}$ is area, $\mathrm {PDP_{N}}$ is power delay product, $\mathrm {V_{th,N}}$ is the threshold voltage and $\mathrm {P_{Leakage,N}}$ is leakage power. All the parameters are normalized(N) to the proposed DM-IST circuit. The FOM of all the considered circuits is listed in Table 3. The FOM of the proposed DM-IST is higher than that of all other considered circuits. From the result it is observed that the FOM of Conventional^14^, Dokic et al.^15^, Anselmno et al.^16^, and Fernandes et al.^17^ are 0.0398$\mathrm {\times }$, 0.9825$\mathrm {\times }$, 0.1189$\mathrm {\times }$ and 0.0519$\mathrm {\times }$ than the proposed DM-IST circuit.

## Conclusion

This work briefly analyses the Dual Mode Inverting Schmitt Trigger (DM-IST) circuit configured with a control unit for dual purposes as an inverter for large voltage range and the Schmitt trigger for high noise immunity. DM-IST has larger hysteresis width, reduced delay, dynamic power. The Monte Carlo simulation of $\mathrm {V_{th}}$ and $\mathrm {V_{LH}}$ and $\mathrm {V_{HL}}$ has reduced variability Thus, DM-IST is efficient and robust to process variation and has improved noise immunity, making it suitable for noisy signal. Further, for reliability analysis, we have studied $\mathrm {V_{th}}$ shift over applying stress for ten years and estimated its process variation and mismatch by running Monte Carlo simulations. The proposed DM-IST is more process-resilient with less $\mathrm {V_{th}}$ shift along with reduced process variation and mismatch. Further, this work has been extended for analyzing the radiation hardening effect by calculating critical charge ($\mathrm {Q_{crit}}$) for all circuits. It is found that the DM-IST has a higher critical charge than other considered circuits. Moreover, the threshold current along with $\mathrm {Q_{crit}}$ has been evaluated for all the considered circuits and compared with the DM-IST.Further, the control unit is tuned over a range of voltages to evaluate $\mathrm {Q_{crit}}$ at different operating conditions. All work has been performed using an industry standard 40nm UMC technology node.

## Data Availability

All data generated or analysed during this study are included in this published article.
